# Ready to ROC? A tutorial on simulation-based power analyses for null hypothesis significance, minimum-effect, and equivalence testing for ROC curve analyses

**DOI:** 10.3758/s13428-025-02646-x

**Published:** 2025-03-18

**Authors:** Paul Riesthuis, Henry Otgaar, Charlotte Bücken

**Affiliations:** 1 Faculty of Law and Criminology, KU Leuven, Leuven, Belgium; 2 Faculty of Psychology and Neuroscience, Maastricht University, Maastricht, The Netherlands

**Keywords:** Smallest effect size of interest, Power analysis, Simulations, Receiver operating characteristic curve, Area under the curve

## Abstract

The receiver operating characteristic (ROC) curve and its corresponding (partial) area under the curve (AUC) are frequently used statistical tools in psychological research to assess the discriminability of a test, method, intervention, or procedure. In this paper, we provide a tutorial on conducting simulation-based power analyses for ROC curve and (p)AUC analyses in R. We also created a Shiny app and the R package “*ROCpower*” to perform such power analyses. In our tutorial, we highlight the importance of setting the smallest effect size of interest (SESOI) for which researchers want to conduct their power analysis. The SESOI is the smallest effect that is practically or theoretically relevant for a specific field of research or study. We provide how such a SESOI can be established and how it changes hypotheses from simply establishing whether there is a statistically significant effect (i.e., null-hypothesis significance testing) to whether the effects are practically or theoretically important (i.e., minimum-effect testing) or whether the effect is too small to care about (i.e., equivalence testing). We show how power analyses for these different hypothesis tests can be conducted via a confidence interval-focused approach. This confidence interval-focused, simulation-based power analysis can be adapted to different research designs and questions and improves the reproducibility of power analyses.

## Introduction

An imperative issue in psychological research is how well a test, method, intervention, or procedure can discriminate between signal and noise. For example, psychologists may be interested in how accurately an inventory assessing depression (e.g., Beck Depression Inventory II; Beck et al., 1961) classifies *depressed* individuals as having depression (i.e., signal; true case) and how often it misclassifies *healthy* individuals as having depression (i.e., noise; false case). Another example could be when psychologists want to measure how eyewitness memory performance is affected by alcohol by examining how well intoxicated and control participants can discriminate between *experienced* (e.g., studied items; signal) and *non-experienced* *events* (e.g., non-studied items; noise; Brady et al., 2023).

A widely used statistical approach to assess discriminability is through the receiver operating characteristic (ROC) curve analysis and its corresponding effect size – the (partial) area under the curve ([p]AUC; Smithson, 2023). To detect a meaningful ROC curve and AUC or meaningful ROC curves and AUC differences between groups (e.g., intoxicated vs sober participants) with sufficient statistical power, it is pivotal to accurately estimate the sample size required through power analyses. However, power analyses for ROC curves and (p)AUC analyses are rarely conducted and even less frequently reproducible when they are reported (e.g., Riesthuis & Otgaar, 2024). This article will provide a tutorial on conducting reproducible simulation-based power analyses for ROC curve and AUC analyses.

To do so, we will first introduce the ROC curve and (p)AUC analyses via an example from the eyewitness memory field. Then, we will introduce how such analyses can be interpreted and explain the role of the smallest effect size of interest (SESOI^1^). The SESOI is the smallest effect size that researchers deem practically or theoretically relevant (Lakens et al., 2018). Establishing the SESOI will change the hypotheses researchers may want to test (Riesthuis, 2024). Specifically, when a SESOI is established, researchers should not just be interested in examining whether, for example, intoxicated eyewitnesses perform statistically significantly worse in discriminating between experienced and non-experienced events (i.e., null-hypothesis significance testing), but also whether this effect is practically or theoretically relevant (i.e., minimum-effect testing; Murphy & Myors, 1999). Moreover, a SESOI makes it possible to examine whether a difference is too small to care about, even if statistically significant (i.e., equivalence testing^2^; Mazzolari et al., 2022; Westlake, 1972). Hence, we will demonstrate how to perform simulation-based power analyses for null-hypothesis significance testing, minimum-effect testing, and equivalence testing for ROC curve and (p)AUC analyses.

## Receiver operating characteristic curve analysis

To illustrate the procedure of a ROC curve analysis, we will provide a simplified example. Imagine that psychologists want to examine whether intoxication can undermine eyewitness memory. To examine the memory undermining effect of alcohol, 100 participants consume alcohol (i.e., experimental group) and another 100 participants receive a placebo (i.e., control group). Subsequently, they watch a video of a mock crime, and after a certain delay, their memory is tested. In line with best practices (see Brady et al., 2023), a confidence-based recognition task is used to assess participants’ memory performance (e.g., “on a scale from 1 to 6, how confident are you that you saw a gun?”; *1* = *very confident that the detail is new, 6* = *very confident* *that the detail is old*). In this memory test, each participant is presented with ten details that were seen in the video (i.e., old items) and ten details that were not (i.e., new items). Thus, in this example, the data consist of 1000 confidence ratings (i.e., 10 old items * 100 participants) for old items and 1000 confidence ratings for new items (i.e., 10 new items * 100 participants) aggregated from all participants for each group (i.e., alcohol and placebo).^3^ The data of this hypothetical experiment might end up looking like the data in Table 1.

**Table 1:** Hypothetical dataset for ROC curve analysis

Alcohol	Very confident new	Somewhat confident new	Not sure—new	Not sure—old	Somewhat confident old	Very confident old	Total number of items
Old items	100	150	300	100	200	150	1000
New items	300	200	100	150	150	100	1000
Placebo							
Old items	50	100	350	150	250	100	1000
New items	350	250	150	100	100	50	1000

Based on these data, the researchers can calculate the true positive rate (TPR) and false positive rate (FPR) at each confidence threshold. The TPR is calculated by dividing the number of old items classified as old (i.e., hits) by the number of old items classified as old plus the amount old items classified as new (i.e., misses):

$$True\;positive\;rate\;(TPR) =\frac{hits}{hits+misses}$$

The FPR is calculated by dividing the number of new items classified as old (i.e., false alarms) by the number of new items classified as old plus the number of new items classified as new (correct rejections):

$$False\;positive\;rate\;(FPR)=\frac{false\;alarms}{false\;alarms+correct\;rejections}$$

For ROC curve analyses, the TPR and FPR are calculated for each confidence threshold and plotted. That is, for the first confidence threshold (i.e., very confident old), it is simply changing the table as if old and new items are only classified as old items when participants indicated a “very confident old” response (see Table 2).

**Table 2:** Contingency table for first confidence threshold

Alcohol	All other confidence levels (i.e., as though recognized as ‘new’)	Very confident old	TPR/ FPR	Total number of items
Old items	850	150	0.15	1000
New items	900	100	0.1	1000
Placebo				
Old items	800	200	0.2	1000
New items	950	50	0.05	1000

*TPR* true-positive rate, *FPR* false-positive rate. TPR is given for the “old items” rows and the FPR is given for the “new items” rows.

For this example, this means that the TPR for the *alcohol* group at confidence level “very confident old” is:

$$TPR = \frac{150}{150+850}= .15$$

and the FPR is:

$$FPR=\frac{100}{100+900}=.10$$

To calculate the TPR and FPR for the second threshold “Somewhat confident old”, the table changes again wherein old and new items are now classified as old items when participants indicate “Somewhat confident old” *and* “very confident old” (i.e., to classify the item as old the associated confidence level should be *at least* “Somewhat confident old”; see Table 3).

**Table 3:** Contingency table for second confidence threshold

Alcohol	All other confidence levels (i.e., as though recognized as ‘new’)	Somewhat confident old + Very confident old	TPR/ FPR	Total number of items
Old items	650	350	0.35	1000
New items	750	250	0.25	1000
Placebo				
Old items	550	450	0.45	1000
New items	850	150	0.15	1000

*TPR* true-positive rate, *FPR* false-positive rate. TPR is given for the “old items” rows and the FPR is given for the “new items” rows.

Hence, for the second threshold “Somewhat confident old” for the *alcohol* group, the TPR is:

$$TPR =\frac{200+150}{200+150+650}= .35$$

and the FPR is:

$$FPR=\frac{100+150}{100+150+750}= .25$$

The TPR and FPR are calculated at each threshold for each group (e.g., alcohol and placebo; see Table 4).

**Table 4:** True- and false-positive rates for the alcohol group

Alcohol group	True-positive rate	False-positive rate
Very confident—old	0.15	0.1
Somewhat confident—old	0.35	0.25
Not sure – old	0.45	0.4
Not sure – new	0.75	0.5
Somewhat confident—new	0.9	0.7
Very confident—new	1	1
Placebo group		
Very confident—old	0.1	0.05
Somewhat confident—old	0.35	0.15
Not sure – old	0.5	0.25
Not sure – new	0.85	0.4
Somewhat confident—new	0.95	0.65
Very confident—new	1	1

**Fig. 1:**
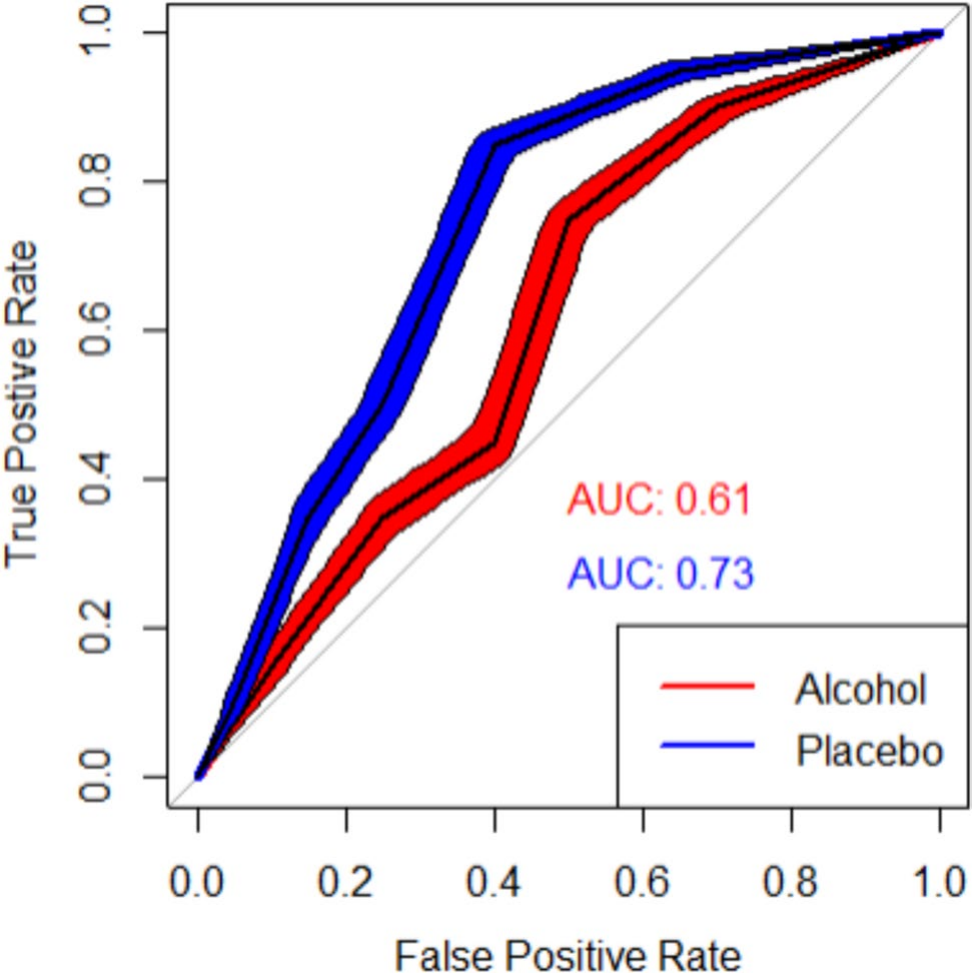
Example of a single ROC curve with the 95% CI along the ROC curve and its associated AUC. See Table 4 for the associated true and false positive rates, which are plotted in this ROC curve. All figures of ROC curves reported in the article were made using “pROC” (Robin et al., 2011) and “ROCpower” in R and the R code can be found at https://osf.io/fv6zh

Then the ROC curves are simply the TPR (on the *y*-axis) and FPR (on the *x*-axis) for each confidence interval (CI) plotted from most conservative (i.e., “very confident—old”) to most liberal (i.e., “very confident—new”) from left to right. The ROC curves with 95% CI and the corresponding AUCs for the hypothetical data of the alcohol (AUC = 0.61) and control groups (AUC = 0.73) are shown in Fig. 1.

**Fig. 2:**
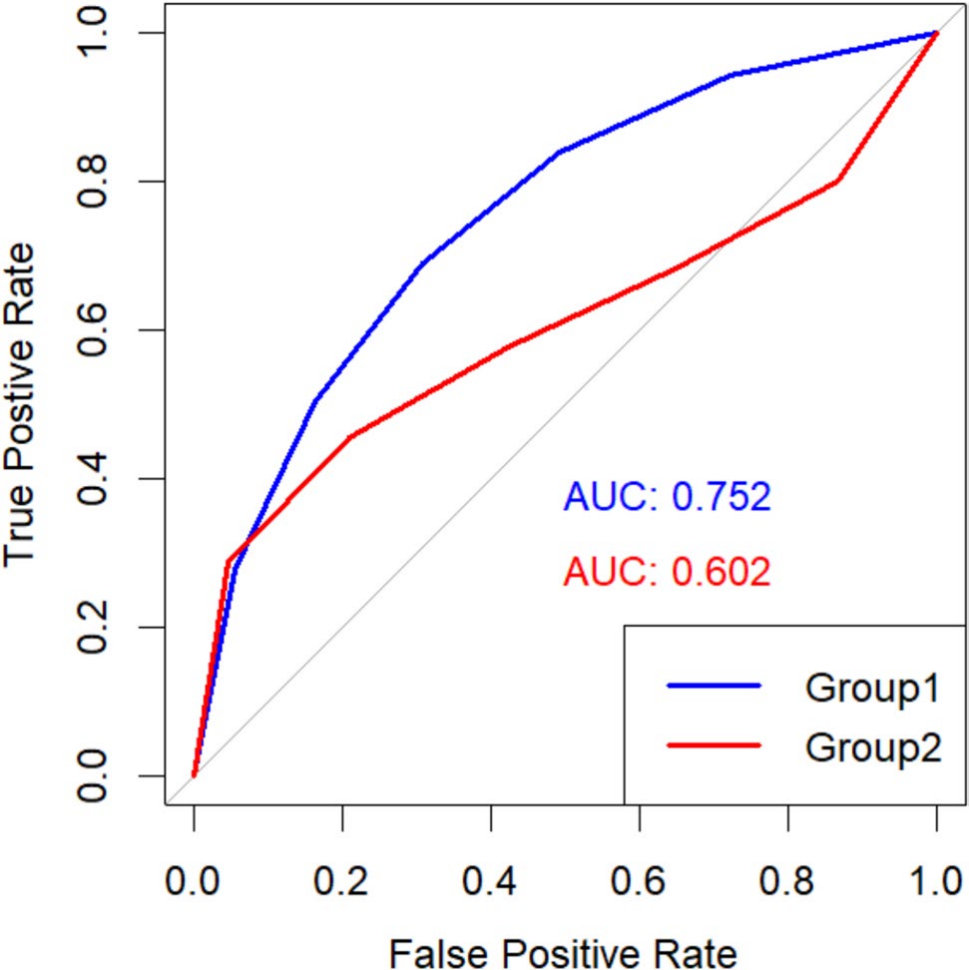
Examples of ROC curves with equal variances (group 1) and unequal variance (group 2)

The shape of the ROC curve depends on the variance of the signal and noise distributions. When the distributions have equal variance, the ROC curve follows a smooth, symmetric shape (see group 1 in Fig. 2). However, if the signal and noise distributions have unequal variance, the ROC curve becomes asymmetric, exhibiting a characteristic bowing effect (see group 2 in Fig. 2).

## Area under the ROC curve

**Fig. 3:**
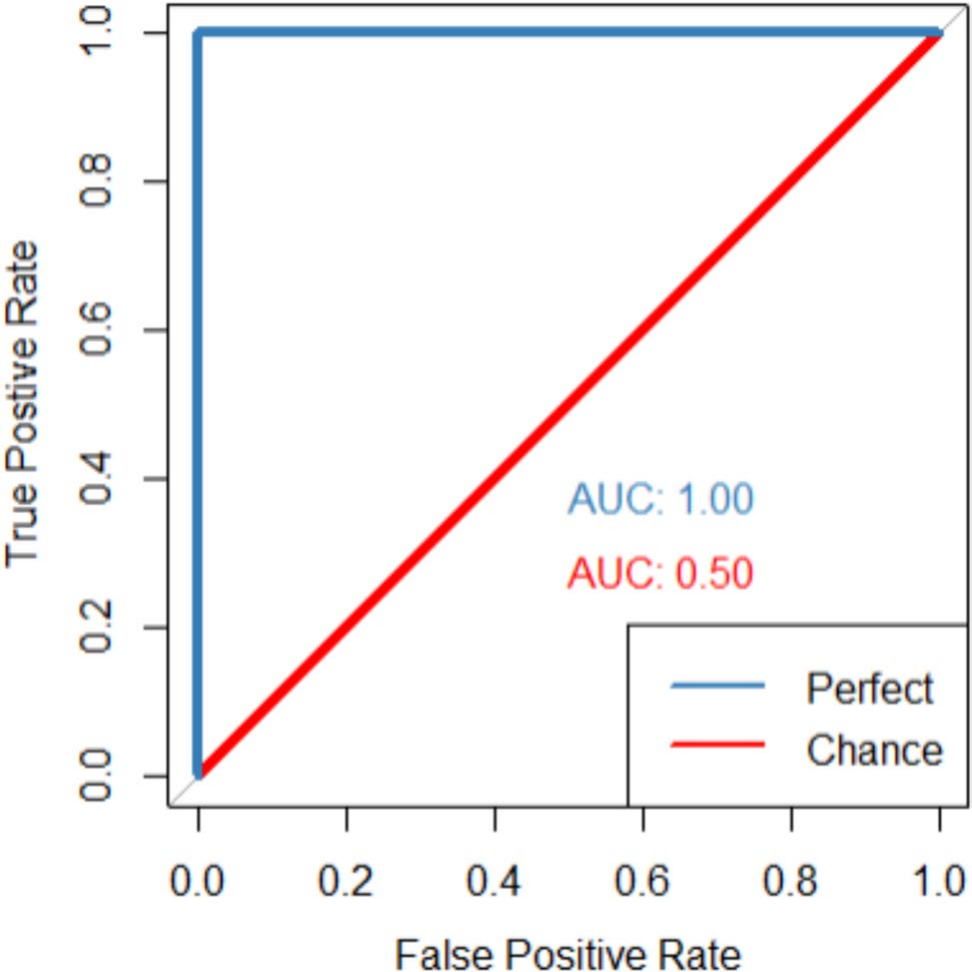
Examples of ROC curves and AUC with perfect (*blue*) and chance-level (*red*) discriminability

When a ROC curve is plotted, the AUC can be estimated, which can be regarded as the effect size (i.e., the magnitude of a phenomenon, Cohen, 1988) of the ROC curve analysis (Ruscio, 2008; Smithson, 2023). The AUC value ranges between 0 and 1 (or 0 to 100%) and higher values indicate better discriminability between signal (e.g., old items) and noise (e.g., new items). An AUC value of 1 indicates perfect discriminability, meaning that all old items are only classified with a “very confident old” response and new items are only classified with a “very confident new” response (see Table 5). This will result in a ROC curve that touches the upper left corner (see blue line in Fig. 2). An AUC value of 0.5 indicates chance-level performance, meaning that participants do not discriminate old items better from new items. An AUC of 0.5 is characterized when responses are equally distributed at each confidence level for old and new items (see Table 5), which results in an ROC curve with a diagonal line (see red line Fig. 3). When conducting ROC curve and AUC analyses of a single test, method, intervention, or procedure, an AUC of 0.5 can be regarded as the null in null-hypothesis significance testing because it indicates that there is no effect (i.e., chance-level discriminability; Youngstrom, 2014). Although the ROC curve typically falls between perfect performance and chance-level performance, the ROC curve can fall below chance-level performance. When the ROC curve is below chance-level performance, it suggests that participants systematically misclassify new items as old and old items as new. Certain manipulations (e.g., type of drug) could lead to such memory-undermining effects. chance-level.

**Table 5:** Data distributions for perfect and chance-level discriminability

Perfect discriminability (AUC = 1)	Very confident—- new	Somewhat confident—new	Not sure—new	Not sure—old	Somewhat confident—old	Very confident—old	Total number of items
Old items	0	0	0	0	0	1000	1000
New items	1000	0	0	0	0	0	1000
Chance discriminability (AUC = 0.5)							
Old items	≈ 167	≈ 167	≈ 167	≈ 167	≈ 167	≈ 167	1000
New items	≈ 167	≈ 167	≈ 167	≈ 167	≈ 167	≈ 167	1000

For chance discriminability, each cell has approximately 167 data points (1000/6 = 166.67). Table can also be changed by having a total number of 1200 items and then each cell has 200 datapoints. It is simply to demonstrate how the chance discriminability data distribution would look.

**Fig. 4:**
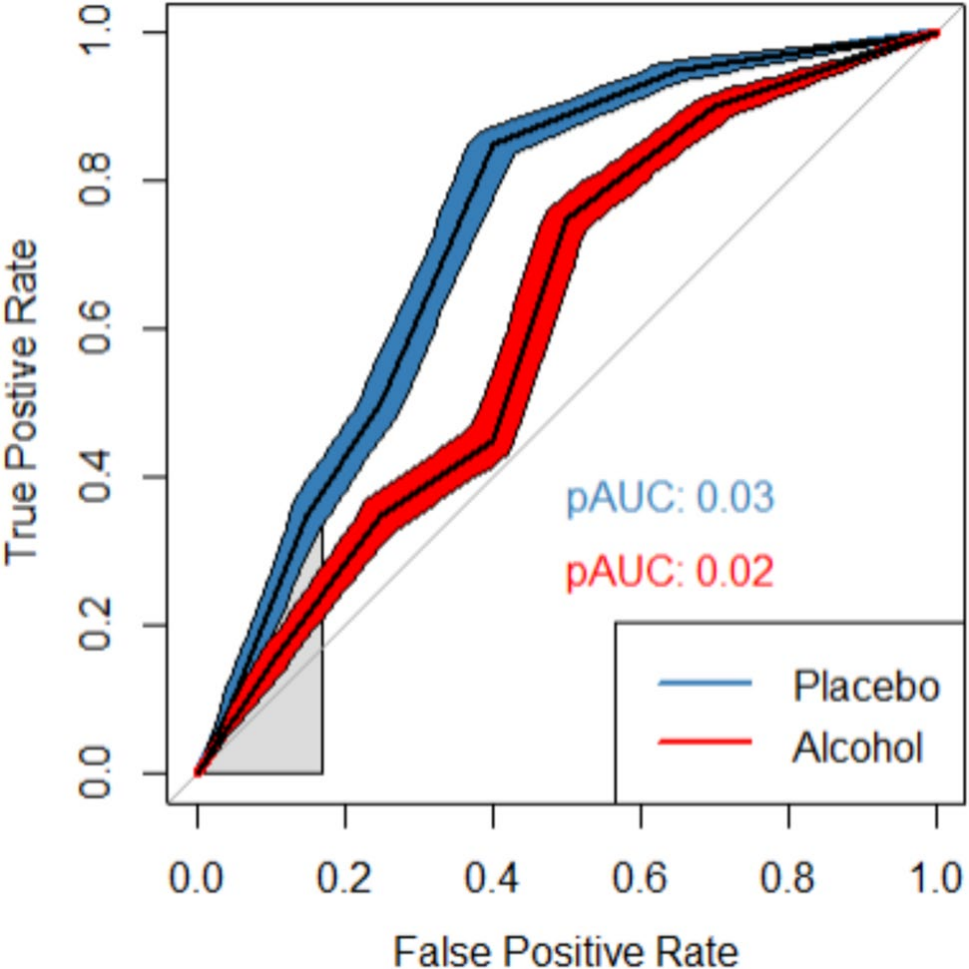
Example of ROC curves for pAUCs for the area under the curve between 0 and .167 with 95% CIs around the ROC curves

It is also possible to estimate the effect size for only a part of the ROC curve, also known as the partial AUC (pAUC). The pAUC can be used when two ROC curves overlap and more localized tests (i.e., examining a specific part of the curve) are necessary to interpret the results (Moise et al., 1988; Obuchowski, 2005; Zou et al., 2007; see Fig. 4). The pAUC is also frequently used in research where the maximum false-positive rate is not 1 but smaller such as in eyewitness identification research where the false positive rate depends on the specific construction of a lineup. That is, in eyewitness identification research, the maximum false-positive rate is oftentimes lower than 1 because only a subset of false-alarm responses are actually scored as a false alarm. For instance, when a fair lineup is constructed with six persons (one innocent suspect and five fillers), the maximum false-positive rate would be 0.167 (1/6) because false alarms are only coded as a false alarm when innocent suspects are identified, and only one of the six lineup members that might be identified is an innocent suspect (i.e., the five others are fillers; Wixted & Mickes, 2018).

## Interpretation of ROC curves and (p)AUCs

There are several ways in which the ROC curves and (p)AUCs can be interpreted. We will highlight how to interpret ROC curves and (p)AUCs using the hypothetical experiment examining the effects of alcohol on eyewitness memory. First, when a ROC curve is plotted, it can be visually examined whether it is above the diagonal line (chance performance; AUC = 0.50). In the case of the hypothetical ROC curve of the alcohol group, the ROC curve was above the diagonal line which suggests that intoxicated participants were able to discriminate between old (studied) and new (non-studied) items above chance-level. To determine whether this ROC curve is statistically meaningful rather than random noise, the 95% CI along the ROC curve should be plotted and examined. Specifically, the lower bound of the CI should be assessed to verify whether it remains above the diagonal line, indicating better than chance-level performance. The associated AUC value of this ROC curve can be interpreted as the estimate of the probability that a randomly chosen old item received a higher confidence score (that it is old) than a randomly chosen new item (Hanley & McNeil, 1982). Hence, for the alcohol group, there was 0.61 probability that a randomly chosen old item received a higher confidence rating (that it is old) than a randomly chosen new item (see Fig. 1). It can then also be examined whether this AUC is statistically significantly greater than chance-level performance (AUC = 0.50) by examining whether its 95% CI does *not* include 0.50 which for the AUC of the alcohol group was the case 0.61, 95% CI [0.59; 0.64], meaning that the AUC was statistically significantly greater than chance-level performance.

It is also possible to compare the memory performance in terms of discriminability (i.e., distinguishing between old and new items) between intoxicated and sober participants by plotting and examining the ROC curves for each group (see Fig. 1). In this case, researchers can visually see that the ROC curve for the placebo group is above the ROC curve for the alcohol group – and thus shows better discriminability. For visual examination of differences between ROC curves, it is recommended to also plot the 95% CIs along the curves (see Figs. 1, 4; Riesthuis & Otgaar, 2024). Visual inspection of the ROC curves is necessary before AUC values are compared because if the curves overlap, direct comparison of AUCs becomes less informative, and local tests of comparisons (e.g., comparing pAUCs) are required (Moise et al., 1988; Obuchowski, 2005; Zou et al., 2007).

If the ROC curve of the placebo group is completely above the ROC curve of the alcohol group, it suggests that intoxicated participants have a worse memory performance in terms of discriminating between studied and new items compared with sober participants. After visually observing a difference in ROC curves, researchers can also examine whether the ROC curves for the alcohol and placebo groups are statistically significantly different from each other (95% CI of AUC difference does not include 0). However, finding a statistically significant effect does not necessarily mean that the effect is meaningful. The reason is twofold: Small sample sizes can lead to unreliable, inflated, and irreplicable statistically significant effects or to statistically non-significant effects that could potentially be practically relevant. In contrast, large sample sizes can lead to statistically significant but trivial effects (Anvari & Lakens, 2021; Bakker et al., 2012). The latter is specifically problematic for studies using ROC curve and AUC analyses in, for example, the eyewitness identification and memory literature, in which it is not uncommon that relatively large sample sizes are collected (Riesthuis & Otgaar, 2024). To avoid questionable interpretations of statistically significant effects, it is necessary to establish when (p)AUCs or differences between (p)AUCs are truly meaningful, on a practical or theoretical level. One way to do so is by establishing the smallest effect size of interest (SESOI; Lakens et al., 2018).

## Smallest effect size of interest and the AUC

To indicate which AUC values are meaningful, benchmarks have been proposed wherein AUC values between 0.7 and 0.8 are considered acceptable, 0.8 and 0.9 are good, and above 0.9 are excellent (Mandreker, 2010). Even though many researchers rely on effect size benchmarks (Correl et al., 2020), it is not recommended to use them to interpret the magnitude of an effect and infer meaning from one’s results. This is because benchmarks fail to take into account the specific context of a study (Cohen, 1988; Panzarella et al., 2021; Riesthuis et al., 2022; White et al., 2023). In other words, there are no universal benchmarks that indicate whether a certain (p)AUC value is good or whether the difference in (p)AUC values between two ROC curves is practically or theoretically meaningful. To establish which (p)AUC values or differences are meaningful for a specific research question, it is necessary to consider the specific context of the study.

The SESOI can be established in various ways. For instance, some have used anchor-based methods wherein participants’ self-reported improvement or decline in terms of emotion served as the anchor for what constituted a meaningful effect on the positive and negative affect schedule (PANAS; Anvari & Lakens, 2021). Another approach is the consensus method, which has been used to estimate the SESOI for the hospital anxiety and depression scale in patients with cardiovascular disease (Lemay et al., 2019; see also Riesthuis et al., 2022). The SESOI for eyewitness memory research was assessed through a cost–benefit analysis wherein the focus laid on possible wrongful convictions caused by eyewitness memory errors (Otgaar et al., 2023; for an extended overview of all methods to set the SESOI see Anvari & Lakens, 2021; Riesthuis, 2024).

Whichever approach is used to estimate the SESOI, it is typically recommended to focus on unstandardized effect sizes (Greenland et al., 1986, 1991; Pek & Flora, 2018; Riesthuis, 2024; Schäfer, 2023). That is, unstandardized effect sizes are easier to interpret than standardized effect sizes because they are in the original units of the measurement instrument. Moreover, unstandardized effect sizes are more robust because they do not depend on the variation (e.g., standard deviation) and are typically easier to calculate than standardized effect sizes (Baguley, 2009). However, for the ROC curve and (p)AUC analyses this would mean focusing on the true- and false positive rates and the associated distributions, which can be difficult to calculate and estimate. Hence, to estimate the SESOI for ROC curve analyses, it may be more feasible to use the effect size AUC (i.e., for a single ROC curve) or differences in AUCs (i.e., for comparing two groups).

Specifically, for a single ROC curve, researchers can set their SESOI by defining the minimum probability at which a randomly chosen signal datapoint (e.g., a studied item or a patient with depression) should yield a higher score (e.g., confidence rating, depression scale score) than a randomly chosen noise datapoint (e.g., a new item or a healthy patient) for the effect to be considered practically or theoretically meaningful. This probability—captured by the AUC—reflects the model’s ability to discriminate between signal and noise. Even though comparisons often involve two groups (e.g., studied items vs new items), a single ROC curve represents the performance of a method, intervention, or procedure across all possible decision thresholds, summarizing how well it distinguishes signal (i.e., studied items) from noise (i.e., new items). For instance, if there is an interviewing technique that can improve eyewitness memory but is difficult to learn and expensive due to the extensive training necessary, larger AUCs (e.g., AUC ≥ 0.80) may be required to deem it practically relevant, considering the costs of implementation. However, if a simple instruction (e.g., only report what you remember) can improve eyewitness memory, lower AUCs (AUCs ≥ 0.60) might be practically relevant because the costs are next to none and the instruction thus only provides benefits. Similar justifications can be made if researchers want to compare different tests, methods, interventions or procedures. Of importance is that the SESOI does not have to be perfect as long as the researchers set and justify their SESOI a priori (Riesthuis, 2024).

## SESOI, hypotheses, and confidence intervals

When researchers have decided upon a SESOI for their study, they can adapt their hypotheses from the typical null-hypothesis significance testing framework to take into account the SESOI (Riesthuis, 2024). Specifically, researchers may not simply want to establish whether an AUC difference is statistically significantly different from 0 (or for single ROC curve analyses different from an AUC of 0.50 – chance-level discriminability), but whether it is greater than the SESOI, also known as minimum-effect testing (Murphy & Myors, 1999). In other words, the chosen SESOI replaces the null. Another possibility with the SESOI is that researchers can examine whether a difference is too small to care about (equivalence testing; Westlake, 1972). In this last example, researchers may want to provide evidence for the null hypothesis (e.g., no meaningful memory differences between intoxicated and sober witnesses) through equivalence testing.

One way to conduct minimum-effect testing or equivalence testing is by using the confidence interval approach (CI; Jané et al., 2024; Riesthuis, 2024; Smiley et al., 2023). CIs can be used to determine whether results are statistically significant (null-hypothesis significance testing), practically or theoretically meaningful (minimum-effect testing), or equivalent (equivalence testing). A recent study showed how such a CI approach can be used to conduct simulation-based power analyses for various research designs (e.g., [in-]dependent sample t-test, correlational studies, and multilevel models; Riesthuis, 2024). Specifically, if the 95% CI of the effect does not include 0, then the effect can be considered statistically significant (*p* < 0.05^4^; null-hypothesis significance testing). Minimum-effect testing is simply an extension of null-hypothesis significance testing wherein the null is replaced by the SESOI and then it is examined whether the 95% CI does not include the SESOI.^5^ For equivalence testing, it is examined whether the 90% CI^6^ is smaller in magnitude than the SESOI (i.e., whether the effect falls within the lower and upper equivalence bounds set based on the SESOI). In the current tutorial, we show how the CI-focused approach can be extended to ROC curve and (p)AUC analyses. To be able to adequately address the different types of hypothesis testing (i.e., null-hypothesis significance testing, minimum-effect testing, equivalence testing), the hypotheses should be reflected in the a priori power analyses to determine how many participants are required for a specific study.

## Power analyses for ROC curve and AUC analyses

To date, there is limited information on how to conduct power analyses for ROC curve and AUC analyses (but see Mah, 2022). This issue is reflected in a recent study by Riesthuis and Otgaar (2024), wherein it was found that out of 239 experiments that used ROC curve analyses in eyewitness memory research, power analyses were conducted only for 65 experiments. The majority of these power analyses were not directly conducted for the ROC curve analyses but for other types of statistical analyses (e.g., independent samples *t* tests, one-way ANOVA, etc.). Moreover, out of the 65 conducted power analyses, only three were fully reproducible with the information provided by authors and 19 were reproducible when certain assumptions were made (e.g., regarding the used alpha level, desired statistical power, directionality of test, etc.). This inability to reproduce power analyses can become problematic. That is, if power analyses cannot be reproduced, it is uncertain whether estimated sample sizes are correct, which could mean that statistical analyses are underpowered (e.g., if the samples are too small to conduct the desired statistical test) to assess the effects of interest or overpowered (e.g., if sample sizes are too big), meaning that trivial and non-meaningful effects might be found (Anvari & Lakens, 2021; McKay et al., 2023).

## Simulation-based power analyses for ROC curve analyses

In the present article, we provide a tutorial on how to conduct reproducible simulation-based power analyses in the statistical software R (R Core Team, 2023) or in a Shiny app (https://paulriesthuis.shinyapps.io/ROC_Power_Shiny/).^7^ We also developed the R package “*ROCpower”* (Riesthuis, 2025; https://github.com/PaulRiesthuis/ROCpower), which provides functions to visualize full and partial ROC curves, compute (p)AUCs, and conduct power analyses for single and two-group (partial) ROC curve and (p)AUC analyses which are the same as the provided R code and Shiny app. The idea behind simulation-based power analyses is straightforward. First, researchers indicate their parameters of interest such as sample size, effect size (i.e., AUC; AUC difference), alpha level, and study parameters (e.g., amount of studied and new items, correlation for paired ROCs) and generate, for example, 1000 datasets based on these parameters. Then, these datasets can be analyzed individually the same way in which researchers would analyze them with their eventual collected data (e.g., conduct ROC curve and AUC analyses). Afterwards, researchers can extract the relevant statistics such as *p* values, 95% CIs, and 90% CIs for the AUC or AUC difference for each analyzed simulated dataset. CIs are calculated via either the DeLong (full ROC curves; DeLong et al., 1988; Robin et al., 2011) or bootstrapping method (partial ROC curves). CIs for the AUC difference are currently only provided for the comparisons of paired ROC curves in the pROC package (Robin et al., 2011). However, CIs for the comparisons of unpaired full ROC curves can be calculated by deriving the standard error (SE) of the AUC difference from the test statistic calculated via DeLong’s (DeLong et al., 1988; Sun & Xu, 2014) or bootstrapping method, which are readily available in the pROC package. Using the SE of the AUC difference (Hanley & McNeil, 1982, 1983), 95% and 90% CIs for the AUC difference can be calculated as follows:

$${CI}_{\Delta AUC}=\Delta AUC \pm {{z}_{(95\%=1.96; 90\%=1.645)}SE}_{\Delta AUC}$$

These can then be used to calculate the statistical power for the provided sample size. Specifically, for a single ROC curve the statistical power for null-hypothesis significance testing is the proportion of results of which the 95% CI does not include the AUC of 0.5 (or 50%). For minimum-effect testing, the statistical power, is the proportion of results of which the 95% CI of the AUC is greater than the SESOI. For equivalence testing, it is the proportion of results of which the 90% CI of the AUC are within the equivalence bounds which are set by the SESOI. For the comparisons of ROC curves, the statistical power for null-hypothesis significance testing is the proportion of results of which the 95% CI of the AUC difference does not include 0, for minimum-effect testing it is the proportion of results of which the 95% CI of the AUC difference does not include the SESOI, and for equivalence testing, it is the proportion of results of which the 90% CI of the AUC difference are within the equivalence bounds which are set by the SESOI.

In this tutorial, we will show how simulation-based power analyses can be conducted for ROC curve and AUC analyses for null-hypothesis significance, minimum-effect, and equivalence testing when a SESOI is set. The steps for the various simulation-based power analyses (e.g., single ROC curve and AUC, two [un-]paired ROC curves and AUC values, etc.) are as follows: (1) decide whether to estimate the (p)AUCs for equal variance (for normal curvilinear ROC curves) or unequal variance (for specific shape of ROC curves) ROC curves, (2) indicate whether the power analysis is for a single (p)ROC curve and (p)AUC or (un)paired (p)ROC curves and difference in (p)AUCs, (3) provide study parameters (e.g., sample size, number of studied and new items), and (4) indicate how many simulations to run (default = 1000) and conduct the simulation-based power analysis. We provide elaborate explanations and examples to show how statistical power can be estimated for a single ROC curve and AUC value, for comparisons between (un)paired ROC curves and AUC values (e.g., to compare the performance between intoxicated and sober witnesses), and for pROC curves and pAUCs. The R code and Rmarkdown files are available on the Open Science Framework (https://osf.io/h2kvj/) and the Shiny app can be accessed here: https://paulriesthuis.shinyapps.io/ROC_Power_Shiny/. To make sure that the simulation-based power analyses are reproducible, users should either share the R code that they used to conduct the power analyses or provide the information that is given in the Shiny app or “*ROCpower*” package in their manuscripts.

## Required software

In this tutorial, we used the following open-source research software: *R* (R Core Team, 2023), *shiny* (Chang et al., 2023), *shinydashboard* (Chang & Borges Ribeiro, 2021), *pROC* (Robin et al., 2011), *MASS* (Venables & Ripley, 2002), *knirt* (Xie, 2024), *kableExtra* (Zhu, 2024), *dplyr* (Wickham et al., 2023), *tidyverse* (Wickham et al., 2019), and *rempsych* (Thériault, 2023).

## Simulation-based power analysis for single ROC curve and AUC

**Fig. 5:**
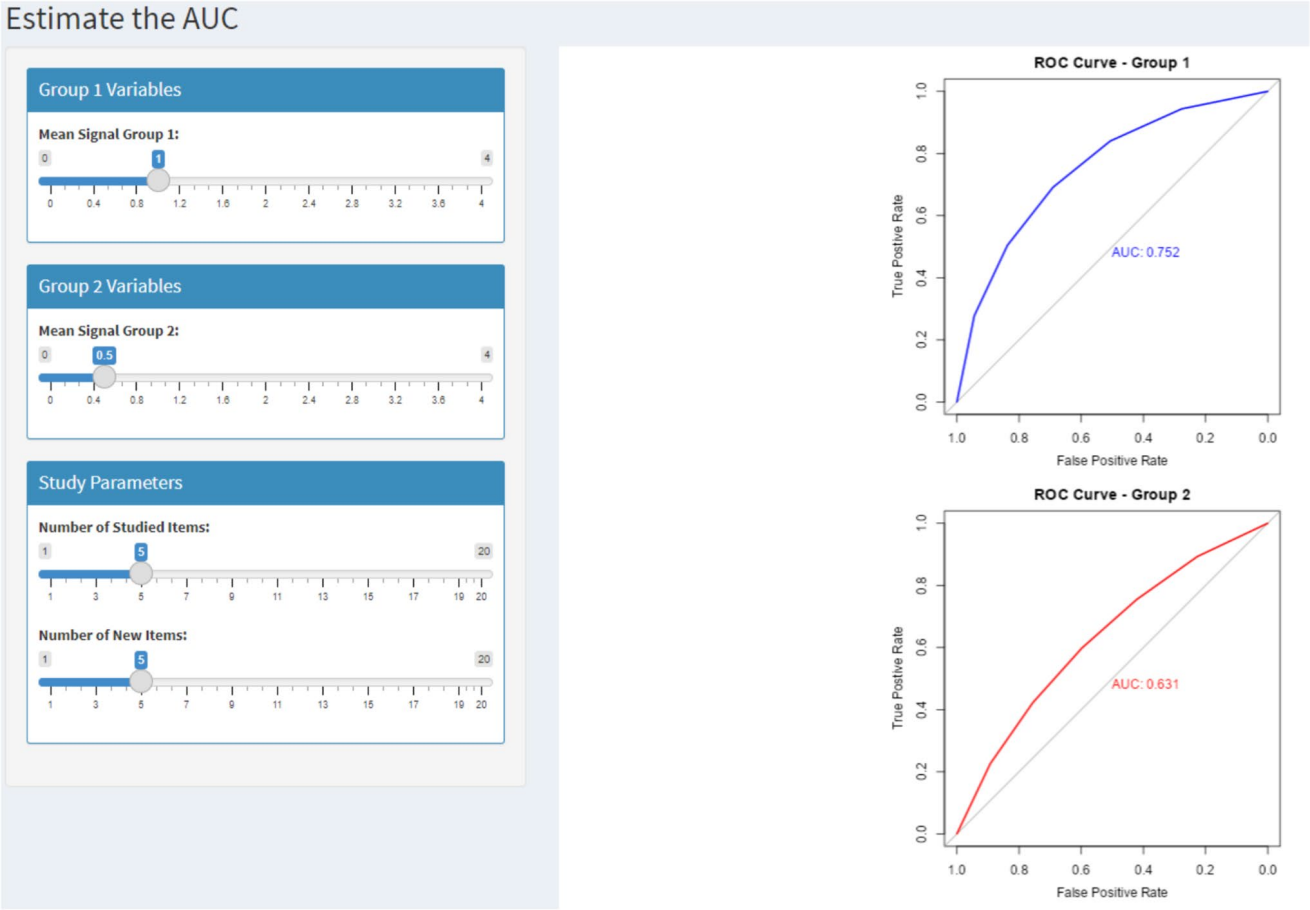
Image of the Shiny app tab 1 “Estimate the AUC – equal variance”, which can be used to estimate the AUCs of two groups

**Fig. 6:**
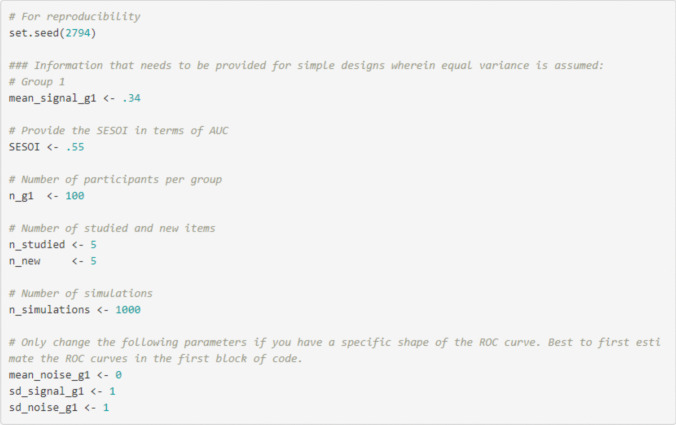
Example of the code for a single ROC curve and AUC in R

We first explain the steps that need to be taken to conduct a simulation-based power analysis for a single ROC curve and AUC analysis and then show how to do it for a hypothetical experiment on the effects of alcohol on memory. To conduct a simulation-based power analysis for a single ROC curve and AUC analysis, researchers first have to estimate the AUC of interest for which the underlying dataset is required. The easiest way to estimate the AUC for a single ROC curve is by changing the mean of the signal distribution of group 1 in the Shiny app tab 1—“Estimate the AUC – equal variance” where the ROC curves and corresponding AUCs are automatically plotted for a sample size of 10,000 to get precise estimates of the AUC for the parameters of interest (e.g., mean of the signal distribution, number of studied items, number of new items; see Fig. 5). The mean of the signal distribution is simply used to create a dataset to estimate the AUC of interest (see below for more information on how the AUCs are estimated and how the associated datasets are constructed). For single ROC curves with equal variance (i.e., normal curvilinear ROC curve) in the Shiny app, the mean of the signal distribution of group 1 in tab 1—“Estimate the AUC – equal variance” is automatically set as the mean of signal distribution in tab 2—“Single ROC curve – equal variance” and the mean of the noise distribution is set to 0 with standard deviations (SD) of the signal and noise distributions set to 1. If researchers want to create ROC curves with unequal variance to adjust the shape of the expected ROC curve and estimate the AUCs, they can adjust the following parameters: mean of the noise distribution, and SDs of the signal and noise distributions in the Rcode or Shiny app (see Fig. 6; or tab 5 “Estimate the AUC – unequal variance” in the Shiny app). It is also possible to visualize the ROC curves using the function “visualize_full_roc()” (or “visualize_partial_roc” for partial ROC curves) of the R package “*ROCpower*”.

The means and SDs of the signal and noise distribution, and numbers of signal (e.g., studied items) and noise (e.g., “new items) items are used to create a dataset on which ROC curve analyses can be conducted and AUCs estimated. Specifically, based on the means and SDs of the signal and noise distribution, and numbers of signal and noise items, random normally distributed values are generated. We generated values from a normal distribution as previous research has shown that estimates of the AUCs are practically unaffected by deviations from normality (see Hajian-Tilaki et al., 1997; Hanley, 1988). These values are then redistributed on a scale from 1 to 6 to reflect the confidence ratings of the experiment that is hypothesized by the researchers (if continuous data are desired, this redistribution step can be skipped in the Rcode). This will provide a dataset with one column indicating the signal (= 1) and noise (= 0) datapoints and another column with the associated confidence ratings (ranging from 1–6). Based on such a dataset, a ROC curve analysis can be conducted via the pROC package (Robin et al., 2011). For a single ROC curve, this means plotting the ROC curve and estimating the AUC and its 95% CIs and 90% CIs.

When the AUC of interest for a single ROC curve is estimated, researchers have to provide the following information in the Rcode or in the Shiny app (i.e., under tab 2 “Single ROC curve – equal variance – power analysis”) to conduct the simulation-based power analysis for an equal-variance ROC curve: mean of the signal distribution (to indicate the AUC of interest; in the Shiny app this is automatically filled in to be the same as the one used to estimate the AUC for group 1 in tab 1), the SESOI in terms of AUC (only for minimum-effect testing or equivalence testing), number of participants, number of studied items per participant (i.e., signal items; in the Shiny app this is automatically filled in to be the same as in tab 1), number of new items per participant (i.e., noise items; in the Shiny app this is automatically filled in to be the same as in tab 1), the number of simulations (Fig. 6; see also tab 2 “Single ROC curve – equal variance – power analysis” in the Shiny app). For unequal variance ROC curves (see tab 5 “Single ROC curve – unequal variance – power analysis” in the Shiny app) the following needs to be provided in addition to the information above: mean of the noise distribution and SDs of the signal and noise distribution.

**Fig. 7:**
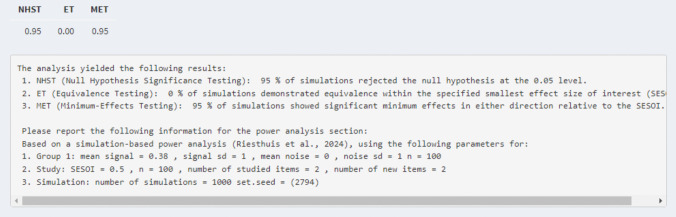
Example of results of statistical power for the hypothetical experiment for each test derived from the Shiny app

Then, based on these parameters, the simulation-based power analysis can be conducted during which 1000 datasets^8^ are created and analyzed individually. For a simulation-based power analysis for a single ROC curve that means the 95% CI and 90% CIs of the AUC for each dataset are calculated. Then, based on the 95% CIs and 90% CIs of the AUC, the statistical power for various hypothesis tests (null-hypothesis significance testing, minimum-effect testing, equivalence testing) can be calculated. Specifically, for null-hypothesis significance testing, the statistical power is the proportion of 95% CIs of the AUC that do not include 0.50, for minimum-effect testing, the statistical power is the proportion of 95% CI of the AUC that is greater than the SESOI, and for equivalence testing the statistical power is the proportion of 90% CIs of the AUC that are smaller in magnitude than the SESOI. In the Rcode, a table with the statistical power for each test is provided. In the Shiny app, the table and text with information concerning the statistical power and reproducibility are provided, as shown in Fig. 7.^9^ The power analysis can also be conducted via the “*ROCpower*” package in R by using the function “simulate_single_roc()”.

## An example

Through a hypothetical experiment on the memory-undermining effects of alcohol, we will show how the simulation-based power analysis for a single ROC curve and AUC analysis can be conducted for the various hypothesis tests (i.e., null-hypothesis significance testing, minimum-effect testing, and equivalence testing). In this hypothetical experiment, researchers want to assess memory performance in terms of discriminability (i.e., distinguishing between studied items and non-studied items) for intoxicated participants. To examine this, researchers will provide participants with alcohol and then have them study a list of items (e.g., words). After a certain delay, the participants will complete a confidence-based recognition task wherein old (e.g., studied; signal) and new (e.g., non-studied; noise) items are presented and they have to indicate on a scale from 1 to 6 how confident they are that the item is new or old (e.g., *1* = *Very confident new, 6* = *Very confident old)*. Each participant will be asked about two old and two new items in this recognition task. Hence, we will show how the researchers can conduct a simulation-based power analysis of the ROC curve and AUC analysis to estimate the statistical power of the various hypothesis tests for different sample sizes and the number of old and new items.^10^

**Fig. 8:**
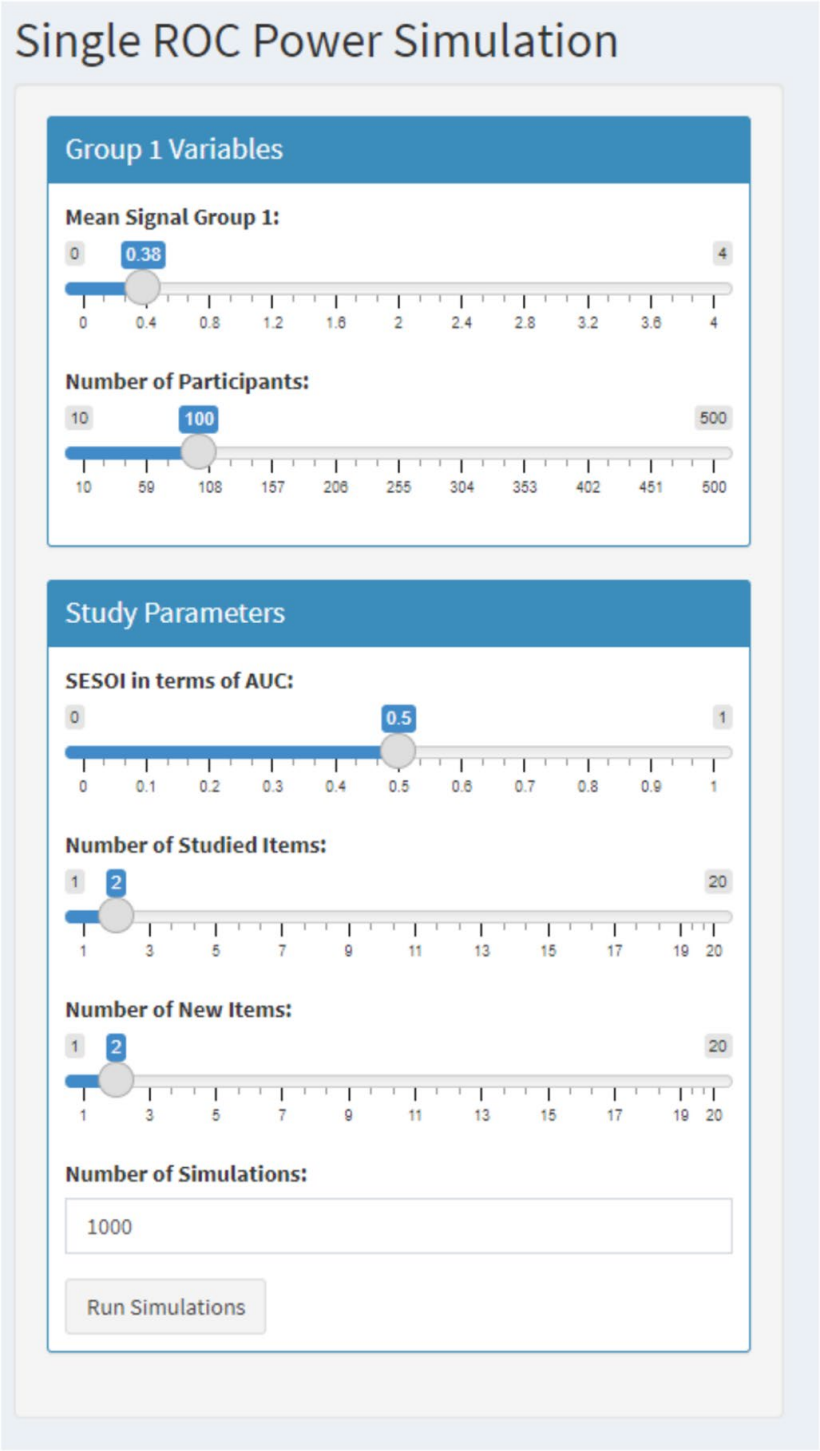
Example of required information to conduct the simulation-based power analysis for a null-hypothesis significance test for a single ROC curve and AUC analysis

*Power analysis single ROC curve and AUC for null-hypothesis significance testing.* Assume that the researchers are interested in an AUC of 0.60 for the alcohol group. The researchers can adjust the mean signal for group 1 to obtain an AUC of 0.60 (i.e., mean of signal distribution = 0.38 for two studied and two new items) and its underlying dataset. Further, they want to know the statistical power for 100 participants in which each participant received two studied and two new items. This is all the required information to conduct the simulation-based power analysis for a null-hypothesis significance test for a single ROC curve and AUC analysis if equal variances are assumed (see tab 2 “Single ROC curve – equal variance”; see Fig. 8). The results indicated that for a power analysis for a null-hypothesis significance test with an AUC of 0.60 (mean of signal distribution = 0.38) with 100 participants who each received two studied and two new items, the statistical power was 0.95 (see Fig. 7).

**Fig. 9:**
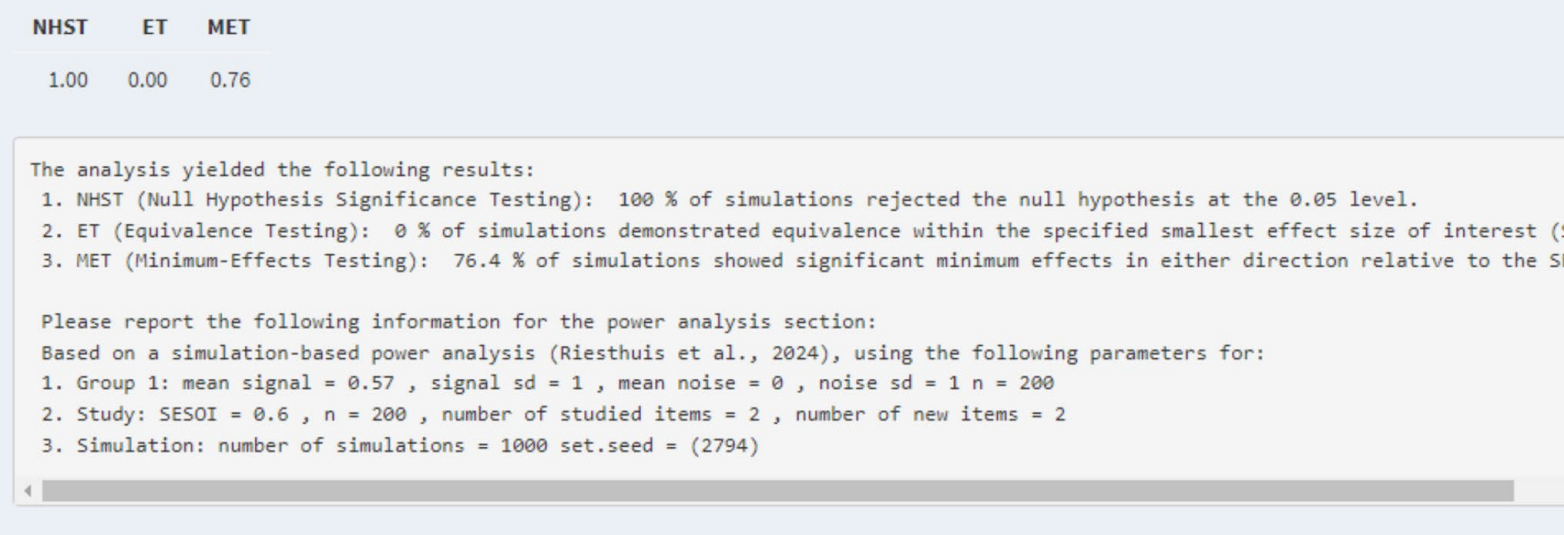
Results of the power analysis for the example of minimum-effect testing for a single ROC curve/AUC

*Power analysis single ROC Curve and AUC for minimum-effect testing.* If researchers want to conduct simulation-based power analyses for a minimum-effect test, they need to indicate what their SESOI is in terms of AUC (e.g., AUC = 0.60). Then, to conduct a simulation-based power analysis for a minimum-effect test, the researchers have to slightly increase the estimated AUC (e.g., adjust the mean of the signal distribution of group 1) so that it is higher than the SESOI. This is because if the estimated AUC is equal to the SESOI, it is treated as if there is no effect and the type 1 error rate is observed (approximately 5% of the results have 95% CIs greater than the SESOI). The reason that this happens is that in minimum-effect testing the SESOI replaces the null. When the estimated AUC is adjusted, the researchers can examine how many additional participants (or increased number of old and new items) and with which AUC they will have sufficient statistical power (e.g., < 0.80) for a minimum-effect test (i.e., how much they need to increase the number of participants or number of old and new items in order to get the desired statistical power for a minimum-effect test in the results of the simulation). As an example, recall the previous scenario in which researchers are interested in an AUC of 0.60 for the alcohol group and set this as their SESOI. They can increase the estimated AUC to 0.65 (adjust mean of the signal distribution from 0.38 to 0.57) and leave the participants (*n* = 100) and number of studied and new items the same (= 2 each) and conduct the simulation-based power analysis. The results show that the statistical power for null-hypothesis significance testing increased to 1.00 and the statistical power for minimum-effect testing is 0.46. If the researchers increase the sample size to 200 participants, the statistical power for minimum-effect testing becomes 0.76 (see Fig. 9). Statistical power can also be increased by increasing the number of studied items (i.e., signal items) and new items (i.e., noise items). Through these steps, researchers can establish the right number of participants they need to test and number of signal and noise items they should include in their study design to be able to detect their SESOI meaningfully by means of a minimum-effect test for their study.

**Fig. 10:**
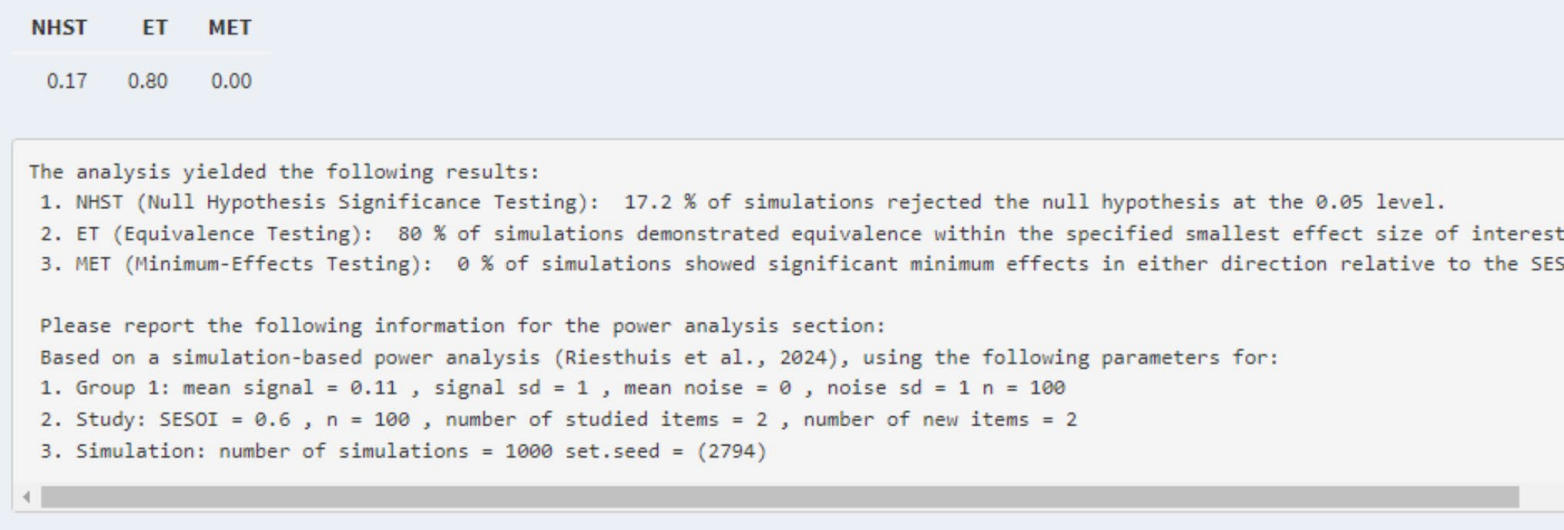
Results of the power analysis for the example of equivalence testing for a single ROC curve/AUC

*Power analysis single ROC curve and AUC for equivalence testing.* For equivalence testing, the researchers also need to indicate what their SESOI is in terms of AUC (e.g., AUC = 0.60). If the estimated AUC is equal to the SESOI, then a type 1 error rate is also observed for equivalence tests wherein approximately 5% of the results have 90% CIs of the AUC that fall within the equivalence bounds set by the SESOI. Hence, for simulation-based power analyses for equivalence testing, researchers should set the estimated AUC to a value smaller in magnitude than their SESOI. For example, they could set their estimated AUC to 0.50 (mean of the signal distribution = 0) or introduce a slightly higher AUC to examine how much statistical power there is to find equivalence even if a small but negligible effect exists. Following the previous example, assume the researchers set the SESOI to an AUC of 0.60 and want to examine the statistical power for equivalence testing using the same parameters as before (*n* = 100, two old items, two new items). They can set the estimated AUC to 0.50 (mean of the signal distribution = 0) and conduct the simulation-based power analysis which will show a statistical power of 0.93 for an equivalence test assuming that there is no effect at all. However, maybe the researchers do think that a small but negligible effect exists. They can change the AUC to, for example, 0.53 (mean of the signal distribution = 0.11), which shows that they still have a statistical power of 0.80 for equivalence testing even if a small AUC exists (i.e., AUC = 0.53; see Fig. 10). Larger estimated AUCs can be indicated but then more participants or number of signal and noise items are required in order to have sufficient statistical power (i.e., > 0.80) for the equivalence test.

## Simulation-based power analysis for two (un-)paired ROC curves and AUCs

Simulation-based power analyses for ROC curve and AUC analyses can also be conducted when groups (paired/within or between/unpaired) are compared. For instance, ROC curves and AUC analyses can be conducted when researchers want to compare the memory performance of intoxicated participants with sober participants (i.e., between-subjects design/unpaired). In these experimental designs, researchers are interested in examining what the difference in ROC curves and AUCs is between the two groups. The steps for simulation-based power analyses for the comparison of two (un-)paired ROC curves and AUCs are very similar to a simulation-based power analysis for the single ROC curve and AUC analysis we conducted in the hypothetical example above. We will first briefly explain the steps that need to be taken for simulation-based power analyses for the comparison of two unpaired ROC curves and AUCs, and then provide an illustrative example via a hypothetical experiment.^11^

As for the simulation-based power analysis for a single ROC curve and AUC analysis, the first step is to estimate the ROC curves and AUCs and the underlying datasets but now for two groups. Again, the easiest way to estimate these is by adjusting the means of the signal distribution for group 1 and group 2 in tab 1—“Estimate the AUC – equal variance” (or for unequal variances in tab 5—“Estimate the AUC – unequal variance”) in the Shiny app (see for example Fig. 5) but the estimation can also be done in R. The datasets and AUCs for each group are generated exactly the same as for a single ROC curve and AUC.^12^ Through the estimation of the AUCs for both groups, the researchers can indicate which difference in AUCs they are interested in.

When researchers have estimated which AUC difference they are interested in, they have to indicate the following information for unpaired equal-variance ROC curves and AUCs: mean of the signal distribution for group 1 (to indicate the AUC of group 1 and the AUC difference), mean of the signal distribution for group 2 (to indicate the AUC of group 2 and the AUC difference), the SESOI in terms of AUC difference (only when using minimum-effect testing and equivalence testing), number of participants in group 1, number of participants in group 2, number of old items per participant (i.e., signal items), number of new items per participant (i.e., noise items), and the number of simulations (see tab 3 “Unpaired ROC curves – equal variance – power analysis” in the Shiny app). For unpaired unequal variance ROC curves the following additional information needs to be provided for both groups: (1) mean of the noise distribution and (2) SDs of the signal and noise distribution (see tab 7 in the Shiny App “Unpaired ROC curves – equal variance – power analysis”).

To conduct the CI-focused, simulation-based power analyses for null-hypothesis significance testing, minimum-effect testing, and equivalence testing for the comparison of two ROC curves and AUCs between the control and experimental group, 95% and 90% CIs of AUC difference are necessary. Then, the steps to conduct the power analysis are similar to those for the single ROC curve and AUC analysis described above, with the only difference being that two ROC curves and AUCs are generated per dataset (i.e., one for each group), and the effect size of interest is the difference in AUCs. Hence, to conduct a simulation-based power analysis for the parameters, 1000 datasets are created and analyzed separately. Based on the 95% CI and 90% CI of the AUC difference, the statistical power for various hypothesis tests (null-hypothesis significance testing, minimum-effect testing, equivalence testing) can be calculated. Specifically, for null-hypothesis significance testing the statistical power is the proportion of 95% CIs of the AUC difference that do not include 0, for minimum-effect testing the statistical power is the proportion of 95% CI of the AUC difference that are greater than the SESOI, and for equivalence testing the statistical power is the proportion of 90% CIs of the AUC difference that are smaller in magnitude than the SESOI. As in the power analysis for a single ROC curve and AUC analysis, the Rcode provides a table with the statistical power for each test and the Shiny app will provide the table and text with information concerning the statistical power and how to make the power analysis reproducible (i.e., which information needs to be reported in a manuscript if this Shiny app was used to conduct the power analysis). The power analysis can also be conducted using the “*ROCpower*” package in R using the function “simulate_two_roc()”.

## An example

We will now provide a hypothetical experiment to show how a simulation-based power analysis is conducted for (un-)paired ROC curves and AUCs analyses. We will use the previously mentioned hypothetical experiment (see power analysis of single ROC curve) on the effects of alcohol on memory. To adapt the design to include two groups, the hypothetical experiment now examines the difference between one group of participants who will consume alcohol (i.e., experimental group) and another group of participants receiving a placebo (i.e., control group). The same confidence-based recognition task will be used wherein participants are asked about two studied items (signal) and two new (noise) items.

**Fig. 11:**
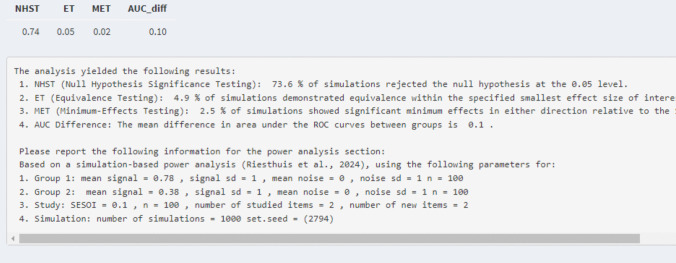
Results of the power analysis for the example of null-hypothesis significance testing for two unpaired ROC curves/AUCs

*Power analysis for two unpaired equal-variance ROC curves and AUCs for null-hypothesis significance testing.* We assume that the researchers are interested in an AUC of 0.60 for the alcohol group and an AUC of 0.70 for the placebo group. Hence, they want to conduct a power analysis for an AUC difference of 0.10. To estimate the AUCs for the two groups they can adjust the mean of the signal distribution for group 1 to 0.38 to obtain an AUC of 0.60 and its underlying dataset and the mean of the signal distribution for group 2 to 0.78 to obtain an AUC of 0.70 and its underlying dataset (i.e., in tab 1 of the Shiny app). Next, they have to provide the following study parameters (i.e., in tab 3 of the Shiny app): 100 participants per group, two studied items and two new items, and 1000 simulations. This is sufficient information to conduct the simulation-based power analysis for a null-hypothesis significance test for two unpaired ROC curves and AUCs if equal variances area assumed (see tab 3 “Unpaired ROC curve – equal variance – power analysis”; see also tab 7 if interested in unequal variances “Unpaired ROC curves – unqual variance – power analysis”). The results indicated that for a power analysis for a null-hypothesis significance test with an AUC difference of 0.10, with 100 participants receiving two studied and two new items, the statistical power was 0.74 (see Fig. 11).

**Fig. 12:**
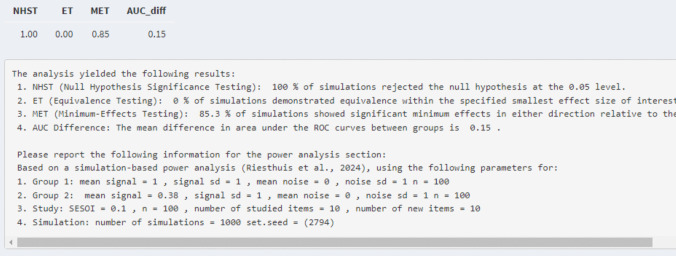
Results of the power analysis for the example of minimum-effect testing for two unpaired ROC curves/AUCs

*Power analysis for two unpaired equal-variance ROC curves and AUC for minimum-effect testing.* If the researchers wanted to conduct the simulation-based power analysis for minimum-effect testing, they have to provide the SESOI. For the current example, we will assume that the researchers set the SESOI of the AUC difference to a 0.10 difference. However, the estimated differences between AUCs cannot be equal to the SESOI because this would lead again to observing the type 1 error rate (SESOI replaces the null in minimum-effect testing). Hence, similar as in the power analysis for the single ROC curve, researchers need to slightly increase the difference between AUCs by adjusting the estimation of the AUCs of the two groups. For instance, the researchers could use an AUC difference of 0.15 instead of 0.10 by increasing the AUC of the control group to 0.75 (mean of the signal distribution of group 2 = 1) while leaving the AUC for the alcohol group at 0.60 (mean of the signal distribution for group 1 = 0.38). This would yield a statistical power for the minimum-effect test of 0.29. To increase the statistical power, the researchers could decide to increase the number of studied and new items to 10 each per participant which would yield a statistical power of 0.85 (see Fig. 12). If researchers prefer not to change the number of items presented, statistical power can also be increased by testing more participants.

**Fig. 13:**
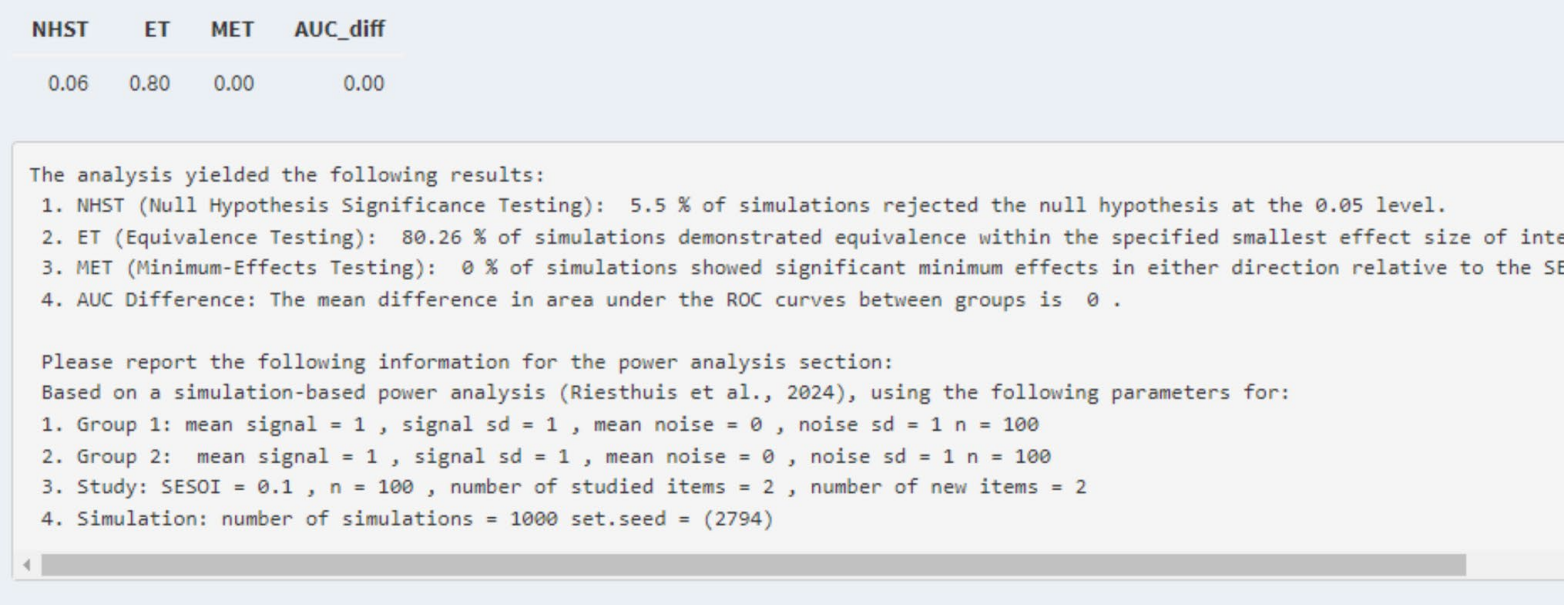
Results of the power analysis for the example of equivalence testing for two unpaired ROC curves/AUCs

*Power analysis for two unpaired equal-variance ROC curves and AUC for equivalence testing.* Last, the researchers can also conduct a simulation-based power analysis for equivalence testing. However, if the estimated AUC difference is equal to the SESOI, then a type 1 error rate is also observed for equivalence testing. So, for simulation-based power analyses for equivalence testing, researchers can set the estimated AUC difference to 0 by changing the mean of the signal distribution of group 1 to the mean of the signal distribution of group 2, or vice versa (to get equal ROC curves and AUCs). It is also possible to examine how much statistical power there is to find equivalence even if a small but negligible effect exists by introducing a small AUC difference (e.g., in this example by setting a difference in AUCs that is > 0 but < 0.10). For the hypothetical experiment, we assume again that the researchers set the SESOI of the AUC difference to 0.10 with the same parameters as before (*n* = 100 per group, two signal items, two noise items) and the means of the signal distribution of group 1 and group 2 are set to be equal (e.g., = 0.1 which leads to an AUC difference of 0). This is sufficient information to conduct the simulation-based power analysis for equivalence testing which will show a statistical power of 0.80 for an equivalence test assuming that there is no effect at all (see Fig. 13).

## Partial ROC curves and partial AUCs

**Fig. 14:**
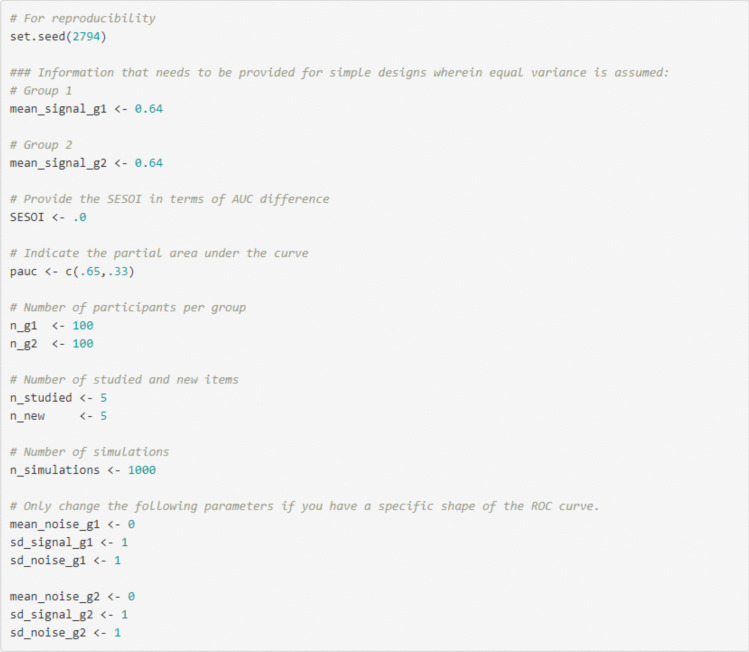
Example of information that needs to be provided to conduct simulation-based power analyses for partial ROC curves and pAUCs

The steps to conduct simulation-based power analyses for partial ROC curves and pAUCs are quite similar for the full ROC curves and AUCs. One difference is that researchers need to provide the SESOI and estimated differences for pAUCs. Moreover, to estimate the pAUC, researchers need to indicate which part of the curve needs to be estimated, which can be done in the Shiny app (see tab 9 in the Shiny App “Estimate the pAUC – equal + unequal variances). However, it is not possible to conduct the simulation-based power analyses for partial ROC curves and pAUCs in the Shiny app. This is because bootstrapping is necessary to examine pAUC differences, which is too computationally heavy for the Shiny app to deal with properly. In fact, a single simulation-based power analysis may take up to 1–2 hours to run (e.g., for 1000 simulations). However, it is possible to conduct the power analysis in R locally by providing the following information and running the simulations directly in R (see Fig. 14). It is also possible to conduct the power analysis using the “*ROCpower*” package in R using the function “simulate_two_partial_roc()”.

## Discussion

ROC curves and their corresponding AUCs are frequently used statistical methods to assess the discriminability of a test, procedure, or method in psychological research. To accurately conduct such analyses and conduct experiments that are appropriately powered to find effects of interest, a priori power analyses should be conducted to estimate how many participants are required. To conduct a priori power analyses, researchers have to decide which effect sizes they are interested in before the study is conducted. Preferably, researchers determine what their smallest effect size of interest (i.e., SESOI) is, so that they can determine which sample size they need to gather to be able to detect meaningful effects (Lakens, 2022; Lakens et al., 2018). However, when a SESOI is set, hypotheses can change, and researchers might not only want to examine whether an effect is statistically significant (i.e., null-hypothesis significance testing) but also whether it is practically or theoretically relevant (i.e., minimum-effect testing), or whether it is too small to care about (i.e., equivalence testing). In this article, we provided a tutorial on how researchers can conduct reproducible simulation-based power analyses for ROC curve and AUC analyses using the CI approach to adequately address various hypothesis tests (i.e., null-hypothesis significance testing, minimum-effect testing, equivalence testing; Riesthuis, 2024; Smiley et al., 2023). We also created a Shiny app and R package (*ROCpower*) to conduct such power analyses.

Our aim with this tutorial is to assist researchers in conducting power analyses for ROC curve and AUC analyses. A reason for developing this tutorial, Shiny app, and R package is because there is scant information on conducting power analyses for these statistical analyses. Specifically, recent research showed that in some fields of psychology (e.g., eyewitness identification and memory), power analyses for ROC curve and AUC analyses were oftentimes not conducted (Riesthuis & Otgaar, 2024). When they were conducted, they were geared towards different statistical tests (e.g., independent sample *t* tests) even though the main analysis of interest was the ROC curve analysis, and were oftentimes not reproducible. When power analyses are omitted or conducted for the incorrect statistical tests, resources may be wasted (Mah, 2022). That is, when too much data is collected it can result in a waste of time and resources but also lead to practical or theoretical claims based on trivial effects (Anvari & Lakens, 2021; Bakker et al., 2012). When researchers rely on heuristics or previous research to estimate sample sizes, it is possible that they collect insufficient data to examine the effects of interest if the resulting sample sizes are too small. It is thus important for researchers to establish which effects they are interested in (i.e., by setting a SESOI) and to accurately power their studies to find these effects.

One way to assess which effects are practically or theoretically relevant is by establishing the SESOI for the specific field of research or study (Lakens et al., 2018; Riesthuis, 2024). Although estimating the SESOI is difficult, researchers are experts in their field and expected to be able to conduct cost–benefit analyses for their specific studies that will initiate discussions on what a possible SESOI could be (Riesthuis, 2024). Important to note is that the SESOI should be established *before* the research is conducted to avoid that researchers will decide which effects are practically or theoretically relevant after results are already known, similar to hypothesizing after results are known (HARKing; Kerr, 1998).

For the simulation-based power analysis, we generated data using a normal distribution for two reasons. First, we wanted to make the simulation-based power analysis as accessible as possible, and through piloting we realized that researchers had difficulties specifying their expected distributions. Second, previous research showed that the estimation of the (p)AUCs of empirical (p)ROC curves are practically unaffected by deviations from normality (Hajian-Tilaki et al., 1997; Hanley, 1988). Hence, we opted to generate data using the normal distribution. We also relied on the frequently used DeLong (1988) or bootstrapping methods to make statistical inferences, which may deviate from what researchers want to implement in their statistical analyses. If researchers have specific predictions about their data and/or the type of statistical analysis they want to conduct, they can adapt the provided R code to conduct the simulation-based power analysis for their specific needs. For instance, if researchers want to take into account the non-normality of their data, they could generate data using the package “latent2likert” (Lalovic, 2024).

## Conclusion

In this tutorial, we showed how to conduct simulation-based power analyses for (p)ROC curves and (p)AUC analyses in R and also created a user-friendly Shiny app. We also developed the R package “*ROCpower*” to facilitate these power analyses. Specifically, through a CI-focused approach, power analyses for null-hypothesis significance tests, minimum-effect tests, and equivalence tests can be conducted. Moreover, the R code and Shiny app for the power analyses can easily be adapted to the researchers’ needs and shared with, for example, co-authors and reviewers, ensuring its reproducibility. Hence, through this tutorial, researchers are ready to conduct power analyses for ROC curve and AUC analyses and appropriately plan for their effect sizes and hypotheses of interest.

## Data Availability

The R code and Rmarkdown files are available on the Open Science Framework (https://osf.io/h2kvj/) and the Shiny app can be accessed here: https://paulriesthuis.shinyapps.io/ROC_Power_Shiny/.

## Footnotes

1 The SESOI is also referred to as the minimally meaningful effect size (MMES; Beribisky et al., 2019) or minimally clinically important difference (MCID; Jaeschke et al., 1989).

2 Also known as negligible effect testing.

3 We used a relatively large dataset (1000 new and 1000 old items) to simplify the example. With a large sample size, the confidence intervals will be narrow and it is important to highlight that if smaller sample sizes are used, the confidence intervals might be larger. That is because confidence intervals are a function of the sample size (Jané et al., 2024).

4 This is based on the traditional α = .05 but this should be justified (see Lakens, 2022).

5 We recommend the use of the 95% CI for two-tailed minimum-effect tests because when the SESOI is relatively small compared to the variation (e.g., standard deviation), using the 90% CI (as in the two one-sided test procedure) inflates the type I error rate to approximately 10%. For one-tailed minimum-effect tests (e.g., tests of superiority and inferiority), 90% CIs should be used.

6 See Alter and Counsell (2023) or Seaman and Serlin (1998) for why 90% CIs are used for equivalence testing.

7 The Shiny app only allows for simulation-based power analyses for full ROC curves and AUC analyses because the pROC and pAUC simulations require bootstrapping, which increases the computational time extensively (approximately 1 h for a simulation of a 1000 datasets). The simulation-based power analyses for pROC and pAUC analyses are provided in the R code and Rmarkdown files.

8 The exact number of simulations can be adjusted in the “Number of Simulations” field in each Power Analysis tab.

9 In the examples in the manuscript we provide power analyses wherein there is an effect. However, all power analyses have been checked on whether the type 1 error rate is inflated in simulation with no effect. All power analyses had a type 1 error rate equal to the significance level.

10 We chose the traditional significance level of 5% and the frequently used 80% statistical power in this article for illustrative purposes. However, the significance level and desired statistical power should be justified (see Lakens, 2022a).

11 The steps for a simulation-based power analysis for paired ROC curves and AUCs is the same except that researchers need to provide a correlation between the signal and noise datapoints. The correlation is ideally based on a realistic value based on earlier studies.

12 For paired ROC curves and AUC analyses, multivariate normal distributions are used to include the correlation.

## Code availability

The R code and Rmarkdown files are available on the Open Science Framework (https://osf.io/h2kvj/) and the Shiny app can be accessed here: https://paulriesthuis.shinyapps.io/ROC_Power_Shiny/.
